# Preclinical proof of concept of a tetravalent lentiviral T-cell vaccine against dengue viruses

**DOI:** 10.3389/fimmu.2023.1208041

**Published:** 2023-08-15

**Authors:** Kirill Nemirov, Pierre Authié, Philippe Souque, Fanny Moncoq, Amandine Noirat, Catherine Blanc, Maryline Bourgine, Laleh Majlessi, Pierre Charneau

**Affiliations:** Pasteur-TheraVectys Joint Lab, Institut Pasteur, Université de Paris, Virology Department, Paris, France

**Keywords:** dengue, DENV, flavivirus, lentiviral vector, ifnar-/-mice, tetravalent vaccine, T-cell vaccine, CD8+ T-cells

## Abstract

Dengue virus (DENV) is responsible for approximately 100 million cases of dengue fever annually, including severe forms such as hemorrhagic dengue and dengue shock syndrome. Despite intensive vaccine research and development spanning several decades, a universally accepted and approved vaccine against dengue fever has not yet been developed. The major challenge associated with the development of such a vaccine is that it should induce simultaneous and equal protection against the four DENV serotypes, because past infection with one serotype may greatly increase the severity of secondary infection with a distinct serotype, a phenomenon known as antibody-dependent enhancement (ADE). Using a lentiviral vector platform that is particularly suitable for the induction of cellular immune responses, we designed a tetravalent T-cell vaccine candidate against DENV (“LV-DEN”). This vaccine candidate has a strong CD8^+^ T-cell immunogenicity against the targeted non-structural DENV proteins, without inducing antibody response against surface antigens. Evaluation of its protective potential in the preclinical flavivirus infection model, i.e., mice knockout for the receptor to the type I IFN, demonstrated its significant protective effect against four distinct DENV serotypes, based on reduced weight loss, viremia, and viral loads in peripheral organs of the challenged mice. These results provide proof of concept for the use of lentiviral vectors for the development of efficient polyvalent T-cell vaccine candidates against all DENV serotypes.

## Introduction

Several highly efficient vaccines have been previously developed against various flaviviruses. Among them, a live-attenuated vaccine against the yellow fever virus (YFV) and inactivated whole virus vaccines against the tick-borne encephalitis virus and the Japanese encephalitis virus induce strong neutralizing antibody responses. Such neutralizing antibodies have frequently been shown to persist for many years, providing long-term protection against their respective diseases (1–4). Studies of protection induced by such vaccines led to the conclusion that neutralizing antibody titers induced by vaccination should be considered as a main correlate of protection. However, in the case of dengue virus (DENV) infections, neutralizing antibody responses often fail to provide protection against the disease (5, 6).

Dengue virus is represented by four distinct serotypes (DENV1-4) that co-circulate in tropical and sub-tropical regions and can repeatedly infect humans. These four DENV serotypes show only 60-75% overall amino acid (aa) similarity. Even though this similarity level is sufficient to induce cross-serotype reactive antibodies, these immune responses are often not completely neutralizing. DENV primarily infects antigen-presenting cells (APCs) that uptake virions, opsonized by antibodies attached to surface proteins, via their Fc receptors for Ig (7–9). Uptake of virions bound to a sufficient quantity of neutralizing antibodies results in their destruction by APCs. However, antibodies generated in response to infection with a given DENV serotype may be less efficient at recognizing other cross-reactive serotypes, resulting in partial or inefficient neutralizing. This results in the uptake of infectious virions by target cells, enhancing DENV infection and ultimately increasing disease severity. This phenomenon is known as antibody-dependent enhancement (ADE) and represents one of the main limitations of DENV vaccine development (5).

To avoid ADE, a prospective vaccine against DENV should induce simultaneous protection against all four serotypes. However, clinical trials of the only currently available tetravalent DENV vaccine, CYD-TDK/Dengvaxia, demonstrated poor protection against DENV-2 despite the high neutralizing antibody titer induced against this serotype (10). Besides, neutralizing antibody titers decrease in the absence of re-exposure in endemic regions, and reinfections with homologous DENV serotypes were reported even in the presence of such antibodies (11). Thus, neutralizing antibody response, considered the best line of defense against many pathogens and a robust correlate of protection for numerous vaccines, does not appear to be sufficiently predictive of the protection against DENV, and may also contribute to more severe disease if the titer of neutralizing antibodies falls below protective level.

Although the precise role of T-cell immunity in protection against DENV is still debated, multiple studies now established that these cellular responses can be protective in both animal models and human populations (12–15). Since mice with functional innate immune responses are highly resistant to DENV infection (16–19), most studies in the field are performed in mice with various types of immunodeficiencies, most commonly on those lacking the receptor to the type I interferons (IFN) (*ifnar^-/-^*) (20–26). The studies performed in these murine models demonstrate that, while protection against homotypic infections is primarily mediated by antibodies, these antibodies are either not protective or enhance heterotypic DENV infections via ADE. In contrast, cytotoxic CD8^+^ T cells were shown to play a significant role in protecting mice against heterotypic DENV infections. Some studies show that although CD4^+^ T cells do not play a direct protective role, they help CD8^+^ T cells in their antiviral effector action (21). Modeling of human CD8^+^ responses in *ifnar^-/-^* mice transgenic for human HLA-B*07:02 allele demonstrated that responses to primary and homotypic DENV infections mainly target serotype-specific epitopes, while responses to heterotypic infections predominantly recognize cross-serotype conserved epitopes. Such epitopes are mostly located in the conserved non-structural (NS) proteins that are highly similar among DENV serotypes (27). Studies of T-cell responses in humans with a history of asymptomatic DENV infections also demonstrated that successive infections with various serotypes induced protective CD8^+^ T cells targeting cross-serotype conserved epitopes mostly located in NS3, NS5, and NS4B proteins (12). NS3 and NS5, as well as the structural protein C, were also identified as major targets of anti-DENV CD4^+^ T cells (28–30). Clinical trials of live-attenuated tetravalent DENV TV003 and TAK-003 vaccine candidates also demonstrated that immunized people develop strong T-cell responses, mostly recognizing cross-serotype conserved epitopes located in NS proteins (14, 31). Altogether, these data suggest that T-cell responses are protective in both humans and mice and could be exploited to develop vaccines triggering broadly protective T-cell responses (11).

More importantly, such a “T-cell vaccine” could overcome the major difficulty associated with ADE, because it would not induce antibody responses against DENV surface proteins that are targeted by non-neutralizing enhancing antibodies. Since protective T-cell epitopes are mainly located in NS proteins that are not implicated in ADE, the vaccine itself would not predispose immunized subjects to enhanced infection post-vaccination due to vaccine-specific antibodies. Moreover, the strong sequence homology among NS proteins from various DENV serotypes can allow the selection of conserved epitopes to be included in a T-cell vaccine for the induction of cross-reactive T cells.

By inducing broadly protective T-cell responses and reducing the probability of serotype-specific antibody responses such a vaccine could limit the number of exposures to DENV surface antigens and minimize detrimental effects of ADE during the formation of protective immune response. A T-cell vaccine will also be particularly useful in circumstances where induction of antibody responses is inefficient or does not provide protection, e.g., due to maternal antibodies interfering with the immunization of infants (32, 33).

Lentiviral vectors represent a particularly promising platform for inducing strong and long-lasting T-cell responses because they directly and efficiently transduce APCs, notably dendritic cells. The endogenous antigen expression in dendritic cells is associated with potent T-cell activation (34). The integration-deficient versions of lentiviral vectors demonstrate efficient antigen presentation without integration of the vector genetic material into the host genome, thus avoiding genotoxicity (35–37). Here we report the development of a non-integrative lentiviral vector-based vaccine candidate that induces significant protection in *ifnar^-/-^* mice against infection with each of the four DENV serotypes. The protective effect is mediated by CD8^+^ T-cell responses directed against conserved regions of DENV NS proteins.

## Materials and methods

### Antigen design

The complete polyprotein sequences of DENV were retrieved from the sequence database (NCBI) (38), aligned with ClustalX (39), and used to construct a phylogenetic tree with Mega 7 software (40). A smaller set of sequences chosen to represent the genetic variability of DENV included five sequences of DENV-1 (GenBank accession N°s: ADC92350.1, AJQ21317.2, ABG75761.1, AIG59667.1, and AAQ19665.2), DENV-2 (ALI16136.1, AAD18036.1, AUZ41807.1, AHA42535.1, and ANT47239.1), and DENV-3 (ACV04798.1, BAE48725.1, AIH13925.1, ALS05358.1, and AIO11765.1), and four sequences of DENV-4 (AVA30162.1, ALI16138.1, AEJ33672.1, and ARN79589.1). MAFFT online service was used to align sequences of known and predicted T-cell epitopes and DENV polyprotein sequences (41). Alignments were visualized with BioEdit sequence editor (42) to further facilitate the selection of epitope-containing regions. Blast search (38) was used to match epitope sequences to the alignment of DENV polyproteins and determine the location of each epitope in the alignment.

Graphic representation of epitope distribution was visualized by XY-plots where each epitope was represented by a single dot showing its position in the alignment on the X axis and the frequency at which it was matched to the alignment on the Y axis. Conserved and/or multiallelic epitopes were identified by a higher matching score and the regions containing such epitopes were preferentially included in DENV poly-antigen.

Major histocompatibility complex class I (MHC-I)-restricted epitope predictions on the IEDB server were performed independently for each of the four DENV serotypes using the Proteasomal cleavage/TAP transport/MHC-I binding combined predictor (43, 44) for the set of 27 most prevalent Human Leukocyte Antigen (HLA) alleles (45). All 8-, 9-, 10-, and 11-mer peptides with a total positive score were retained and combined in a single peptide pool. Predictions on the DTU Bioinformatics server were done using the netCTLpan tool (46) for 9-mer peptides predicted to bind to the 20 most prevalent HLA alleles and retaining those with a consensus rank of less than or equal to 1.0.

The distribution of known and predicted T-cell epitopes was compared and conserved regions containing the maximal number of such epitopes were selected. A 75% majority consensus sequence of each DENV genotype, as well as a master consensus sequence representing all four genotypes — that served as a base for DENV poly-antigen — were created using the Consensus Maker software tool available at the Los Alamos HIV database website (47).

Consensus sequences corresponding to the chosen polyprotein fragments were assembled together as a linear polyprotein and then epitope predictions were repeated to verify that all T-cell epitopes located close to the junction sites were predicted to form correctly and that no junctional non-specific immunodominant epitopes were artificially created. If such epitopes were identified, a de-optimization strategy was applied where hydrophobic amino acid linkers (aa linkers) were inserted at the junction site, followed by additional rounds of epitope prediction, until such non-specific epitopes were no longer predicted.

### Production and titration of lentiviral vectors

A DNA sequence encoding the designed DENV poly-antigen (DEN) and codon-optimized for the expression in mammalian cells was synthesized by GeneScript Biotech (Netherlands) and inserted into pUC57 subcloning vector. The insert was excised on BamHI and XhoI restriction sites and re-cloned into pFLAPΔU3-β2m-WPRE vector (48) between the beta 2 microglobulin (β2m) promoter (48) and the mutated Woodchuck Posttranscriptional Regulatory Element (mWPRE) in which the ATG starting codon was mutated to avoid transcription of the downstream truncated ‘‘X’’ protein of Woodchuck Hepatitis Virus, in order to improve the vector safety. After re-cloning, the sequence of the insert was verified by sequencing (Eurofins, Ebersberg, Germany). Plasmids used for vector production were purified using the NucleoBond Xtra Maxi EF Kit (Macherey Nagel), resuspended in Tris-EDTA Endotoxin-Free buffer, quantified with a NanoDrop 2000c spectrophotometer (Thermo Fisher Scientific), aliquoted, and stored at -80°C.

Lentiviral vectors were produced in Human Embryonic Kidney (HEK)-293T cells, as previously described (49). Briefly, lentiviral vector particles were produced by transient calcium phosphate co-transfection of HEK293T cells with (i) the transfer vector plasmid (pFLAPΔU3-β2m-mWPRE, where the specific antigen was inserted, (ii) an envelope plasmid expressing G glycoprotein of VSV from either Indiana (IND) or New Jersey (NJ) serotype, and (iii) a packaging NDK plasmid. A version of this plasmid bearing a mutation that encodes a D64V substitution in the lentiviral vector integrase was used for the production of non-integrative vectors. Supernatants were harvested 48h post-transfection, clarified by centrifugation at 2500 rpm at 4°C, and ultracentrifuged at 22000 rpm during 1h at 4°C to concentrate vector particles. Pellets were resuspended in sterile 20 mM PIPES buffer pH 7.2, supplemented with 2.5% glucose and 75 mM NaCl, aliquoted, and stocked at −80°C.

The lentiviral vector titer was determined by qPCR on vector-transduced HEK-293T cells that were treated with aphidicolin to prevent cell division. In parallel, HEK-293T cells were transduced with a heat-inactivated (30 min at 70°C) vector to control for plasmid contamination in vector preparation. After 48-72h of transduction, cells were lysed, genomic DNA was isolated, and viral titers were determined by qPCR. To determine the titers, a fragment of lentiviral Flap region was amplified by use of forward 5′– TGGAGGAGGAGATATGAGGG –3′ and reverse 5′– CTGCTGCACTATACCAGACA –3′ primers, as well as a fragment of a cellular GAPDH gene by use of forward 5′– TCTCCTCTGACTTCAACAGC–3′ and reverse 5′– CCCTGCACTTTTTAAGAGCC –3′ primers. The number of vector copies per cell was determined as a ratio of the number of Flap copies to the number of GAPDH copies, which corresponded to the total number of HEK-293T cells. Prior to the immunization of the mice, lentiviral vectors were diluted to the appropriate concentration in PBS.

### Mice

*Ifnar1*-/- mice carry *Ifnar1tm1Agt* allele on either 129 (A129) or C57BL/6J (IFNAR-BL6) genetic background. Both lineages belong to the same MHC haplotype (H-2^b^) and thus have the same antigenic presentation and T-cell response. The initial assessment of LV-DEN immunogenicity and protection efficiency was performed in A129 mice because they were previously shown to be infectable by related Zika flavivirus as well as some DENV strains (35, 50, 51). However, a comparison of DENV infections in A129 and IFNAR-BL6 mice demonstrated that IFNAR-BL6 mice are more susceptible to DENV ((51) and our unpublished data). Although certain symptoms of infection, e.g., ruffled fur, were observed only in A129 mice, the level of weight loss and viremia/viral load of DENV in the organs was higher in IFNAR-BL6 mice. Such difference in infectivity could be particularly important for the analysis of protection against non-mouse adapted strains of DENV and thus most experiments were performed on that mouse lineage. All mice were bred and maintained under specific pathogen-free conditions at the central animal facilities of Institut Pasteur. All experiments involving DENV strains were performed in the A3 isolator unit of the Institut Pasteur animal facility. Experiments on animals were performed in accordance with the European and French guidelines, after approval by the Institut Pasteur Safety, Animal Care and Use Committee (protocol agreement delivered by local ethical committee: CETEA no. DAP1800077) and Ministry of High Education and Research (APAFIS#18428-2019010717408411_v2).

### Immunization and DENV inoculation

For immunization experiments, male and female mice aged 6-18 weeks were used. Immunization of A129 mice was performed either with 5 × 10^7^ TU of integrative lentiviral vector (iLV) or various doses of non-integrative vector (LV), depending on the experiment. Except when indicated, lower doses of LV were used for prime immunizations (1-5 × 10^7^ TU), while higher doses (1-3 × 10^8^ TU) were used for boost or single-dose immunizations. In all experiments, doses of the control LV-GFP vector were identical to the doses of experimental vectors. All immunizations of IFNAR-BL6 mice were performed with 3 × 10^8^ TU of LVs. Immunization was performed by intra-muscular (i.m.) injection of LV in the posterior muscle with a total volume of 50µL.

Inoculations of DENV were performed intravenously (i.v.) via the caudal vein with a total volume of 150µL. The inoculations dose of each DENV serotype depended on the efficiency of its production in Vero E6 cultures. The infectivity of the practicable dose was first verified in preliminary experiments in *ifnar-/-* mice. Mice were monitored for signs of illness, such as lethargy, ruffled fur, and hunched posture, and weight was recorded regularly. Mice were considered to have reached the humane endpoint if they lost 20% of their initial weight.

### Propagation and titration of viral stocks

DENV-1 KDH0026A strain was kindly provided by Dr. Lambrechts (Institut Pasteur, France). Mouse-adapted DENV-2 S221 strain was kindly provided by Dr. Shresta (La Jolla Institute for Allergy and Immunology, La Jolla, CA, USA). DENV-3 PaH881/88 and DENV-4 ThD4_0087_77 strains were both isolated in Thailand in 1988 and 1977, respectively. Virus stocks were produced in Vero E6 cells grown in T-175 tissue flasks with filter cups. Titrations were performed on Vero E6 cells grown on 24 well plates. To do so, cells were inoculated with 300µl of serial stock dilutions for 1 h with periodic shaking. After removal of the inoculation medium, cells were overlayed with DMEM containing 1.6% carboxymethyl cellulose, 2% of heat-inactivated fetal calf serum (FBS), and 1% penicillin/streptomycin. At day 5 post-inoculation, the overlaying medium was removed, cells were fixed with 4% paraformaldehyde for 30 min, and viral foci were revealed by staining with 0.5µg/mL of 4G2 mouse-anti-DENV envelope protein antibody, produced by the recombinant protein production facility of the Institut Pasteur, followed by the second staining with goat anti-mouse IgG conjugated with horseradish peroxidase (HRP) (BioRad, France). The HPR signal was revealed with the Vector VIP peroxidase substrate kit (Vector Laboratories, USA) following the manufacturer’s recommendations.

### ELISPOT assay

ELISPOT kits for IFNγ detection (Mabtech AB, Nacka Strand, Sweden) were used according to the manufacturer’s instructions. Splenocytes from immunized mice were added in triplicates at 1 × 10^5^ cells/well and stimulated with the relevant peptide pools containing 2 μg/ml of each peptide. Unstimulated splenocytes and splenocytes stimulated by 2.5 μg/ml of concanavalin A were used as negative and positive controls, respectively. After 18 h incubation, spots were revealed according to the manufacturers’ protocol and counted with AID ELISpot Reader System ELR04 (Autoimmune Diagnostika GmbH, Strassberg, Germany). Background signals originating from the wells containing unstimulated cells were subtracted and results were expressed as the number of spot-forming cells per million of splenocytes.

### Cytometry

Analysis of intracellular cytokine secretion was performed as described previously (37). Briefly, splenocytes were obtained by tissue homogenization of spleens through 100µm nylon filters (Cell Strainer, BD Bioscience), plated at 4 × 10^6^ cells/well in a 24-well plate, and incubated for 6 h with 10µg/ml of either pooled specific peptides or with equal amount of control non-specific peptide in the presence of 1 mg/mL of anti-CD28 and 1 mg/mL of anti-CD49d monoclonal antibodies (mAbs) (BD Biosciences). During the last 3 h of incubation, cells were treated with a mixture of Golgi Plug and Golgi Stop (BD Bioscience) in the presence of PE-Cy7-anti-CD107a mAb (clone 1D4B, BioLegend). Cells were then washed with PBS supplemented with with 3% FBS (FACS buffer) and incubated for 25 min at 4°C with a mixture of FcγII/III receptor blocking anti-CD16/CD32 (clone 2.4G2), Near IR Live/Dead (Invitrogen), PerCP-Cy5.5-anti-CD3ϵ (clone 145-2C11), eF450-anti-CD4 (clone RM4-5, eBioscience), and BV711-anti-CD8 (clone 53-6.7) mAbs (BD Biosciences or eBioscience). Cells were washed, permeabilized by the use of the Cytofix/Cytoperm kit and incubated with a mixture of PE-anti-IL-2 (clone JES6-5H4), FITC-anti-TNF (MP6-XT22), and APC-anti-IFNγ (clone XMG1.2) mAbs (BD Biosciences), during 30 min at 4°C.

Alternatively, for the analysis of cytokine expression by splenocytes before and after DENV infection, APC-eF780-anti-CD3ϵ (clone 17A2, eBioscience) and APC-anti-CD8 (clone 53-6.7, eBioscience) were used instead of the corresponding mAbs listed above. After incubation with mAbs cells were washed and fixed with Cytofix (BD Biosciences) overnight at 4°C. To study expression of T-cell activation/migration markers, FcγII/III receptor blocking anti-CD16/CD32 (clone 2.4G2, BD Biosciences), FITC-anti-CD8 (clone 53-6.7, Millipore), eF450-anti-CD4 (clone RM4-5, eBioscience), APC-eF780-anti-CD3ϵ (clone 17A2, eBioscience), AF700-anti-CD62L (clone MEL-14, BD Biosciences), and PE-anti-CD44 (clone IM7, eBioscience) were used. Samples were incubated with appropriate mAb mixtures for 25 min at 4°C, washed with FACS buffer, and fixed with Cytofix (BD Biosciences) for 20 min at 4°C. Cells were acquired in an Attune NxT cytometer system (Invitrogen) and data analysis was performed using the FlowJo software (Treestar, OR, USA).

### Determination of viremia and viral loads in peripheral organs

Blood samples used for the analysis of viremia were collected from individual mice by bleeding from the sub-mandibular vein into Microvette 500 K3E EDTA-containing tubes (Starstedt) and centrifuged at 5000g for 10 min in order to separate plasma from blood cells. Clarified plasma samples were kept at -80°C before the RNA extraction followed by RT-qPCR analysis.

RNA was extracted from 35µl of plasma with the QIAamp viral RNA mini kit (QIAGEN, Hilden, Germany). For the analysis of viral load in peripheral organs, a whole organ was collected, weighted, and frozen at -80°C until the moment of RNA extraction. For the extraction, frozen tissue samples were suspended in 1ml of TRIzol (Fisher Scientific) and homogenized in a FastPrep-24 homogenizer (VWR, France) at 6.0 m/s for 30 seconds. Total RNA was purified following the extraction protocol of the TRIzol manufacturer. RNA concentration was determined by Nanodrop spectrophotometer, adjusted to 0.1µg/µl, and 1 µg of total RNA, contained in 10 µl, was used in the RT-qPCR reaction. Two-step RT-qPCR adapted from a previous study (52) was used to determine the viral load in the serum and peripheral organs. The RT reaction was performed by the use of Moloney murine leukemia virus (M-MLV) reverse transcriptase and the resulting product was used in duplicate qPCR run on a QuantStudio 12K Flex real-time PCR system (Applied Biosystems, Carlsbad, CA, USA). Samples originating from mice inoculated with DENV-4 were analyzed using the same RT-qPCR protocol (52), but the reverse primer (5’-ACCAATCCATCTCGCGGCGCT-3’) and TaqMan probe (5’-[FAM]AACATCAATCCAGGCAC[MGBEQ]-3’) were matched to the sequence of DENV-4 strain used for the challenge.

### T-cell depletion *in vivo*

Anti-mouse CD8α (clone 2,43), anti-mouse CD4 (clone GK1.5), and IgG2b control isotype (LTF-2) rat antibody (all from InVivoMab) were used in the T-cell depletion experiments.

### Statistical analysis

Statistical analysis was performed with the statistical tests implemented in GraphPad Prism 9.5.0 (53). For pairwise comparisons, an unpaired parametric t-test with or without Welch’s corrections, depending on the standard deviation (SD) determined from the dataset, was used. Comparative analysis of small sample groups was performed using an unpaired non-parametric Mann-Whitney test. Multiple comparisons were performed either using a one-way ANOVA test (ordinary or Welch, depending on standard deviation) or the Kruskal-Wallis test. Data were considered significant when p-values were less than 0.05.

## Results

### Design of a T-cell DENV poly-antigen

**Figure 1:**
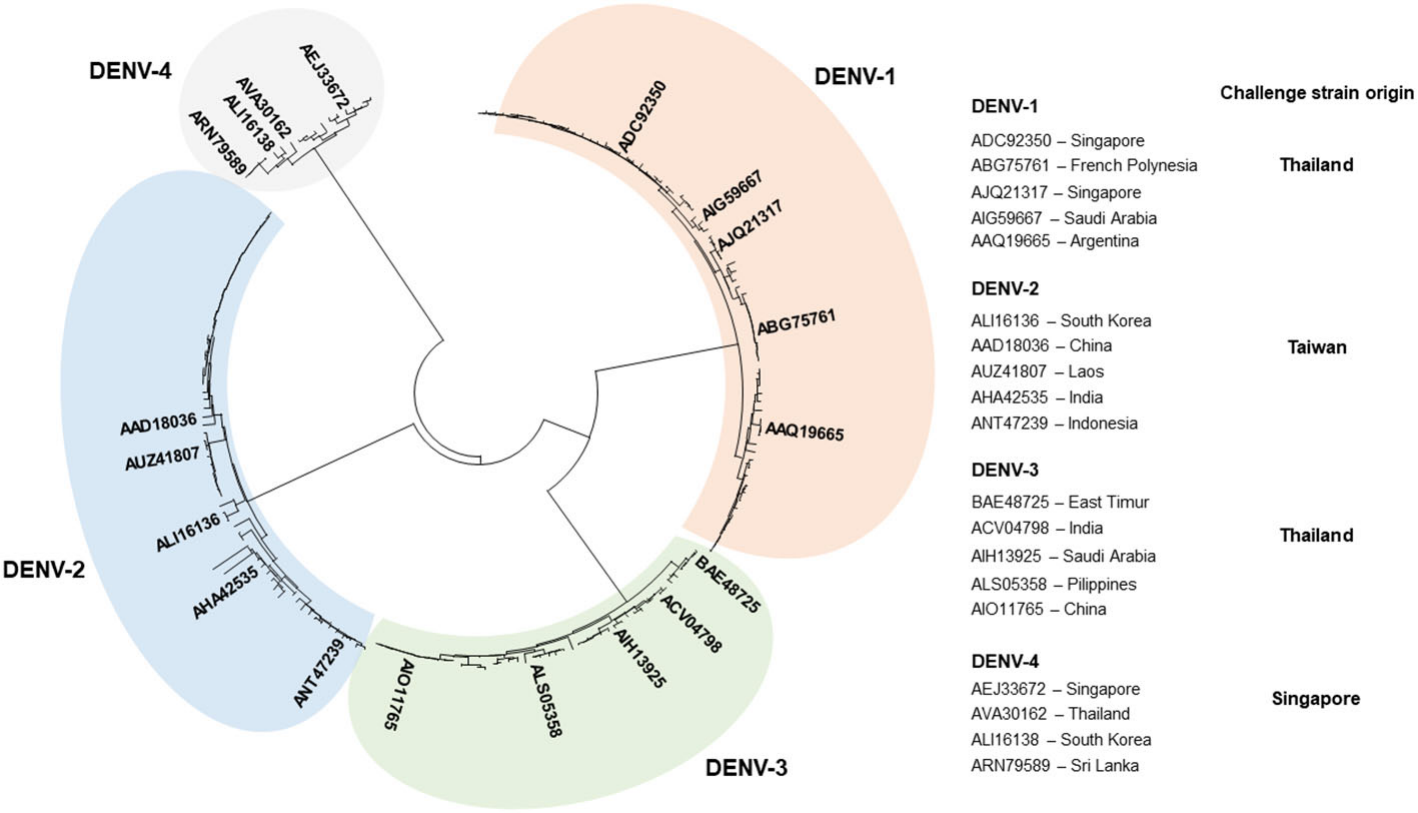
Genetic diversity of DENV. Phylogenetic tree based on the complete polyprotein sequences of DENV-1 (84 sequences), DENV-2 (71 sequences), DENV-3 (46 sequences), and DENV-4 (39 sequences) constructed by use of MEGA 7 software (40). Strains representing distinct phylogenic lineages of each genotype, selected to identify and predict MHC-I epitopes, are shown on the right. “Challenge strain origin” specifies countries where DENV strains used for experimental infection were originally isolated.

**Figure 2:**
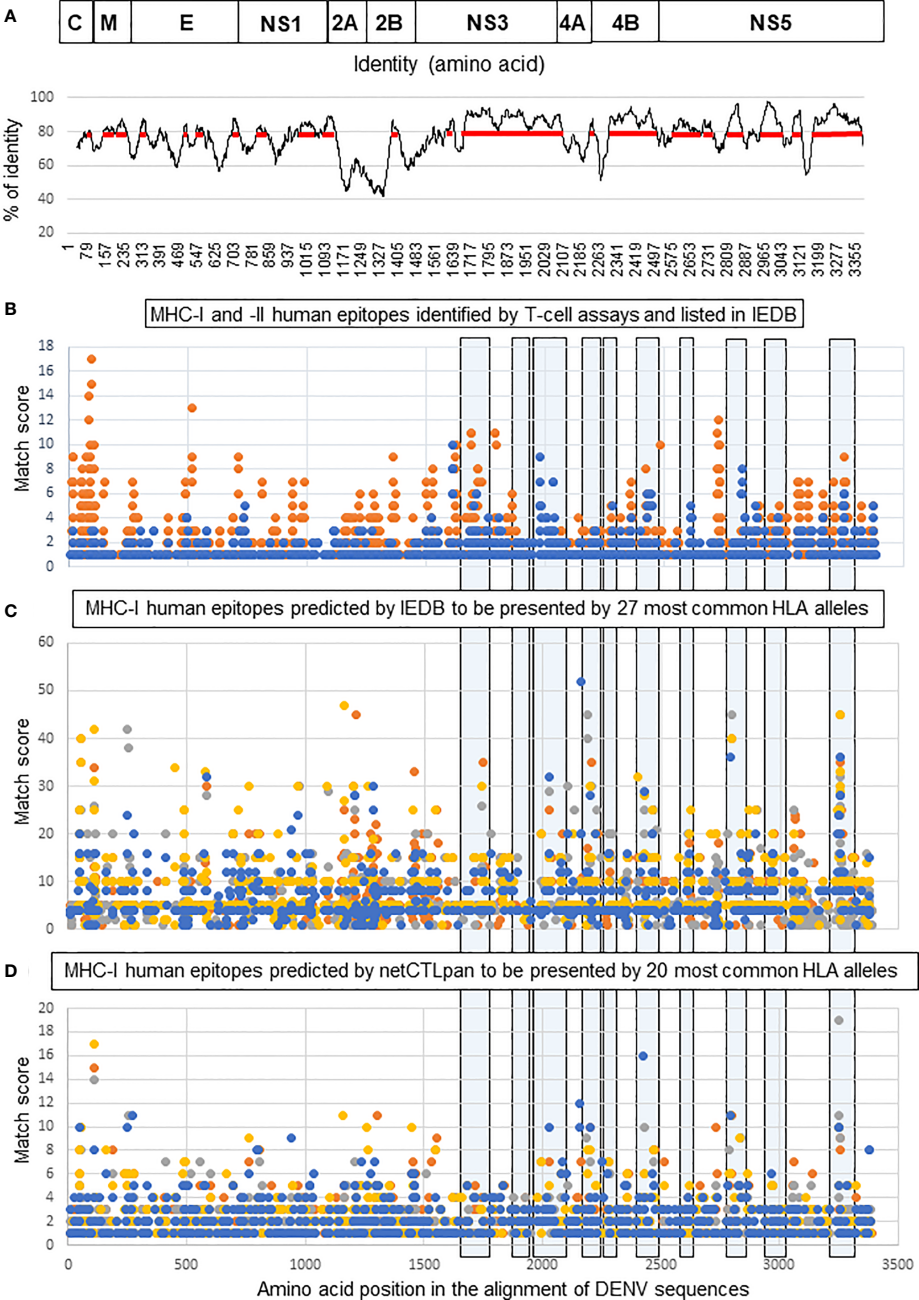
Selection of T-cell epitope-containing regions for DEN poly-antigen design. **(A, top)** Schematic representation of DENV polyprotein. Structural proteins: capsid (C), matrix (M), and envelope (E); and non-structural proteins: NS1, NS2A, NS2B, NS3, NS4A, NS4B, and NS5. **(A, bottom)** Identity plot showing the distribution of identical amino acids in consensus sequences of the four DENV serotypes. The red line shows the regions with an identity score >80%. **(B)** Distribution of human MHC-I (blue) and MHC-II (orange) DENV-specific epitopes, referenced as positive in various T-cell assays in the IEDB database. **(C, D)** Distribution of human HLA class I epitopes predicted for four DENV serotypes by IEDB **(C)** and netCTLpan **(D)** prediction tools. Each dot corresponds to the center of an epitope and its position along the sequence of DENV polyprotein (x-axis). The y-axis indicates the number of times that each epitope could be matched to the alignment of DENV sequences. For instance, y = 5 for each epitope predicted in all DENV-1, DENV-2, and DENV-3 used sequences, and y = 4 for such an epitope in DENV-4. High values on the y-axis correspond to either multiallelic epitopes, i.e., presented by >1 allele, or clustered epitopes, i.e., those that have an identical position on the x-axis. Epitopes predicted for each genotype are shown in different colors: orange (DENV-1), grey (DENV-2), yellow (DENV-3), and blue (DENV-4). Light blue boxes outline the regions selected to create the DEN poly-antigen.

To design a poly-antigen to be used in a tetravalent DENV vaccine candidate, a phylogenetic tree was first constructed using 240 complete polyprotein sequences of the four DENV serotypes derived from the nucleotide sequence database (38). The tree was used to select a smaller set of DENV sequences in which distinct phylogenetic sub-lineages of each serotype were represented by a single sequence (**Figure 1**). Mapping of the regions conserved between distinct DENV serotypes, performed by plotting the similarity score along the polyprotein length, demonstrated that most conserved regions were located in the NS3, NS4B, and NS5 proteins (**Figure 2A**). To select T-cell epitope-containing regions in DENV protein sequences, all HLA class I- or II-restricted epitopes, so far annotated in the Immune Epitope Database and Analysis Resource (IEDB) as positive in T-cell assays, were aligned to the representative DENV sequence set (54).

The distribution of T-cell epitopes along DENV sequence alignment was visualized by an XY-plot (**Figure 2B**) that allowed to identify: (i) epitope-containing regions (epitope clusters) and (ii) individual epitopes, either present in multiple sequences (conserved epitopes) and/or presentable by multiple HLA alleles (multiallelic epitopes). In accordance with published studies, most of the known functionally characterized HLA class I epitopes were located in NS3, NS4B, and NS5, while HLA class II epitopes were more evenly distributed between structural and NS proteins. To improve T-cell epitope mapping, computer prediction of HLA class I epitopes was done using the prediction tools available at IEDB and Technical University of Denmark (DTU Bioinformatics) websites. Predicted epitopes were mapped to the alignment of DENV sequences and visualized by XY-plots (**Figures 2C, D**). Combining the so-far known HLA class I epitopes and those predicted by the two different methods, we selected immunogenic regions to generate a poly-antigen, namely “DEN”. No HLA class II epitope prediction was performed because the methods used to predict such epitopes are less efficient compared to those used for MHC-I epitope prediction (55). Besides, studies in animal models suggest that CD8^+^ T cells play more important roles than CD4^+^ T cells in protection against DENV (20, 21, 23, 25, 26). We thus prioritized the inclusion of the MHC-I-containing regions in DEN poly-antigen. However, as those regions also contain many known MHC-II epitopes (**Figure 2B**), a significant proportion of such epitopes was included in the antigen.

To incorporate genetic variability presented by the four DENV genotypes in a single sequence, a 75% majority consensus was inferred for each DENV genotype, and a master consensus sequence was created based on the four individual consensus sequences (**Figure S1**). In general, the DEN poly-antigen sequence was identical to the master consensus, except for a number of positions where variability was equally split between different genotypes, e.g., S^1674^ of the NS3-1 region in DENV-1 and DENV-3 versus A^1674^ in DENV-2 and DENV-4 or at sites where more significant variations were observed, such as in position 1928 of the NS3-2 region. In such cases, the chosen amino acid corresponded to the most represented residues in the known or predicted T-cell epitopes. Although such an approach was generally applicable, in some short regions the amino acid variation was too substantial to be represented by a single consensus sequence. Therefore, three additional short sequences, i.e., NS3-1A, NS3-3A, and NS3-3B, were included in the DEN poly-antigen to account for this variability (**Figure S1**). The selected regions were joined together, and junction regions were further optimized by inserting aa linkers to remove non-specific epitopes at junction sites (**Figure S2**).

### T-cell immunogenicity of a lentiviral vector encoding the DEN poly-antigen

**Figure 3:**
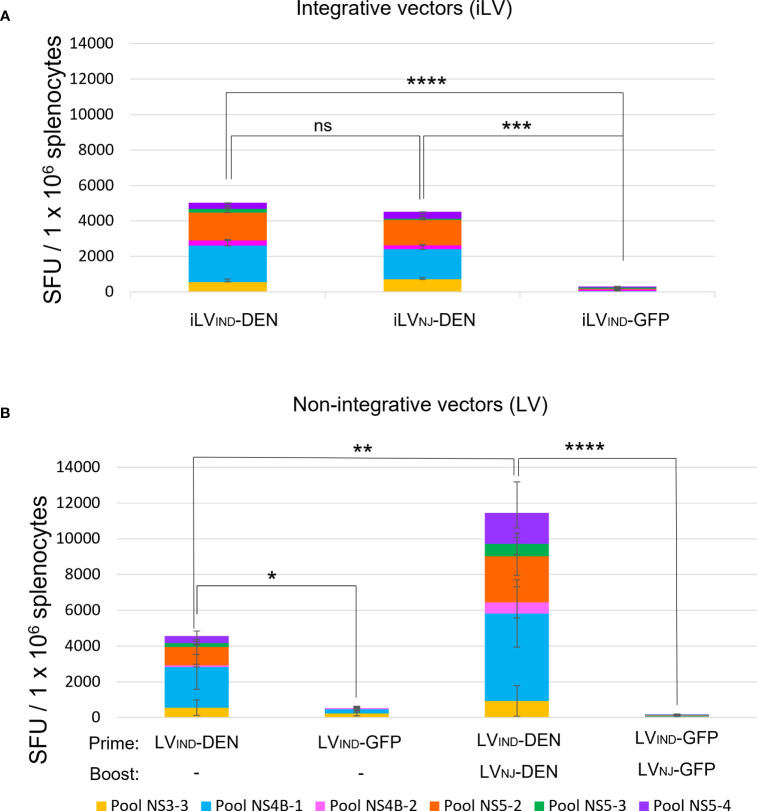
Immunogenicity of integrative (iLV) and non-integrative (LV) lentiviral vectors expressing DEN poly-antigen in A129 (H-2^b^) mice. **(A)** T-cell response induced by two distinctly VSV-G-pseudotyped iLV_IND_-DEN or iLV_NJ_-DEN. A129 (H-2^b^) mice (n = 5/group) were immunized i.m. with iLV_IND_-DEN, iLV_NJ_-DEN, or the control iLV_IND_-GFP. Splenocytes of individual mice were analyzed by IFNγ ELISPOT at day 14 post-immunization, after stimulation *in vitro* with pools of 15-mer peptides, overlapping by 11 amino acids (aa) covering the predicted DEN immunogenic regions for H-2^b^ mice. SFU = spot-forming units. Colored histogram bar segments show median SFU obtained following stimulation with different peptide pools. **(B)** T-cell responses induced by non-integrative LV-DEN after a single immunization or a prime-boost regimen. Splenocytes of individual mice immunized i.m. with LV_IND_-DEN (*n* = 7) or LV_IND_-GFP (*n* = 5) were analyzed by IFNγ ELISPOT at day 14 post-immunization. For the prime-boost immunization, mice (*n* = 6/group) were primed i.m. with LV_IND_-DEN and LV_IND_-GFP and were then boosted i.m. 2 months later with LV_NJ_-DEN and LV_NJ_-GFP, respectively. Splenocytes from individual mice were analyzed by IFNγ ELISPOT on day 6 post-boost. The statistical significance of the total responses was determined by a one-way ANOVA test with Tukey corrections for multiple comparisons (*p < 0.05, **p < 0.01, ***p < 0.001, and ****p < 0.0001).

T-cell immunogenicity of DEN poly-antigen was first analyzed in the context of iLV. In the perspective of a future prime-boost immunization regimen, iLVs were pseudo-typed with envelope glycoprotein from vesicular stomatitis virus (VSV-G) of two distinct serotypes, i.e., Indiana “iLV_IND_-DEN” and New Jersey “iLV_NJ_-DEN”, to avoid that a possible anti-vector immunity induced by the prime could reduce the efficiency of the boost (34, 56). A129 mice (H-2^b^) (*n* = 5/group) were immunized i.m. with a single dose of 5 × 10^7^ TU of iLV_IND_-DEN, iLV_NJ_-DEN, or a negative control vector encoding the irrelevant green fluorescent protein (iLV_IND_-GFP). At day 14 post-immunization, splenocytes were analyzed by IFNγ ELISPOT in response to stimulation with pools of 15-mer peptides overlapping by 11 amino acids, and covering the predicted immunogenic regions for H-2^b^ mice (**Figure S2** and **Table S1**). Immunization with both iLV_IND_-DEN and iLV_NJ_-DEN induced strong multi-specific IFNγ T-cell responses against DEN poly-antigen. No significant differences due to pseudo-typing with VSV-G from IND or NJ serotypes were observed (**Figure 3A**).

Since, for safety reasons, only non-integrative lentiviral vectors, hereafter called (“LV”), can be used as vaccine candidates (34, 56), all following studies were performed with the non-integrative vectors. To establish the T-cell immunogenicity of DEN poly-antigen in the context of a non-integrative lentiviral vector, A129 mice were immunized with a single dose of LV_IND_-DEN (*n* = 7) or LV_IND_-GFP (*n* = 5) (**Figure 3B**). Using various antigens, we have previously established that 10 times more LVs than iLV are necessary to reach the same immunogenicity in different preclinical models (36, 56, 57), which was also applied to the dose of LV used in the present study. Splenocytes from individual mice were analyzed at day 14 post-immunization. With this adapted LV dose (3 × 10^8^ TU), LV_IND_-DEN induced T-cell responses with comparable magnitude to those induced by iLV-DEN (**Figures 3A, B**).

To compare the magnitude of T-cell responses induced by a single dose of LV-DEN versus a prime-boost regimen, A129 mice (*n* = 6/group) were primed i.m. with LV_IND_-DEN or LV_IND_-GFP and boosted i.m. 2 months later with LV_NJ_-DEN or LV_NJ_-GFP, respectively. At day 6 post-boost, a significant increase in the magnitude of T-cell response was observed in the boosted mice compared to those immunized with a single dose (**Figure 3B**).

### Polyfunctionality of T cells induced by LV-DEN

**Figure 4:**
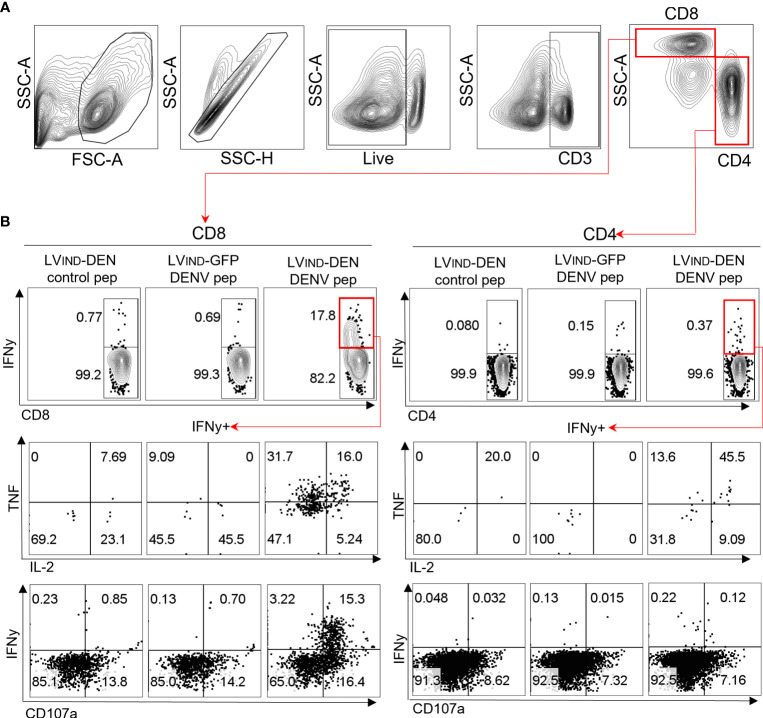
A single LV-DEN injection induces robust polyfunctional CD8^+^ T-cell responses. IFNAR-BL6 mice were injected i.m. with LV_IND_-DEN or LV_IND_-GFP (*n* = 2/group) at day 0. At day 14, splenocytes were collected, pooled by group, and analyzed by ICS. **(A)** Gating strategy to identify CD8^+^ and CD4^+^ T cells. **(B)** Identification of CD8^+^ (left panel) or CD4^+^ T cells (right panel) expressing IFNγ (upper panels), co-expressing IFNγ and TNF/IL-2 (middle panels), or co-expressing IFNγ and the degranulation marker CD107a (lower panels) among each spleen T subset. Splenocytes were stimulated either with an irrelevant control peptide (left) or with a pool of 11 DEN-specific peptides (**Table S1**) derived from immunogenic regions of NS3-3, NS4B-1, NS4A, NS5-2, and NS5-4 (center and right).

Previous studies demonstrated that protective immunity to DENV is associated with the release of IFNγ, TNF, and IL-2 by polyfunctional T cells (20, 58). We performed intracellular cytokine secretion analysis on the splenocytes of *ifnar-/-* C57BL/6 (IFNAR-BL6) mice, which are more susceptible to flaviviral infections than A129 mice and were, therefore, used for the subsequent immunization-protection experiments ((51) and our unpublished data). Pooled splenocytes from mice (*n* = 2/group) immunized i.m. with a single dose of 3 × 10^8^ TU of either LV_IND_-DEN or LV_IND_-GFP were stimulated at day 14 post-injection, either by a pool of 11 DEN-derived peptides or by the same amount of a negative control peptide (**Table S1**), and then subjected to intracellular cytokine staining (ICS) (**Figure 4**). The analysis detected antigen-specific single positive (IFNγ^+^), double positive (IFNγ^+^ TNF^+^ or IFNγ^+^ IL-2^+^), and triple positive (IFNγ^+^ TNF^+^ IL-2^+^) CD8^+^ T cells in the spleen of LV_IND_-DEN-immunized mice but not in the control LV_IND_-GFP-injected group (**Figures 4A, B** top and middle). Moreover, sizeable proportions of IFNγ^+^ CD8^+^ T cells from LV-DEN-immunized mice co-expressed the lymphocyte degranulation marker CD107a (**Figure 4B** bottom), indicating the potential effector functions of these cells. In contrast, only low levels of cytokine production were observed in peptide-re-stimulated CD4^+^ T cells of the LV-DEN-immunized mice (**Figure 4**).

### Protective potential of a single dose of LV-DEN against challenge with DENV-1, -2, -3, or -4 serotype

**Figure 5:**
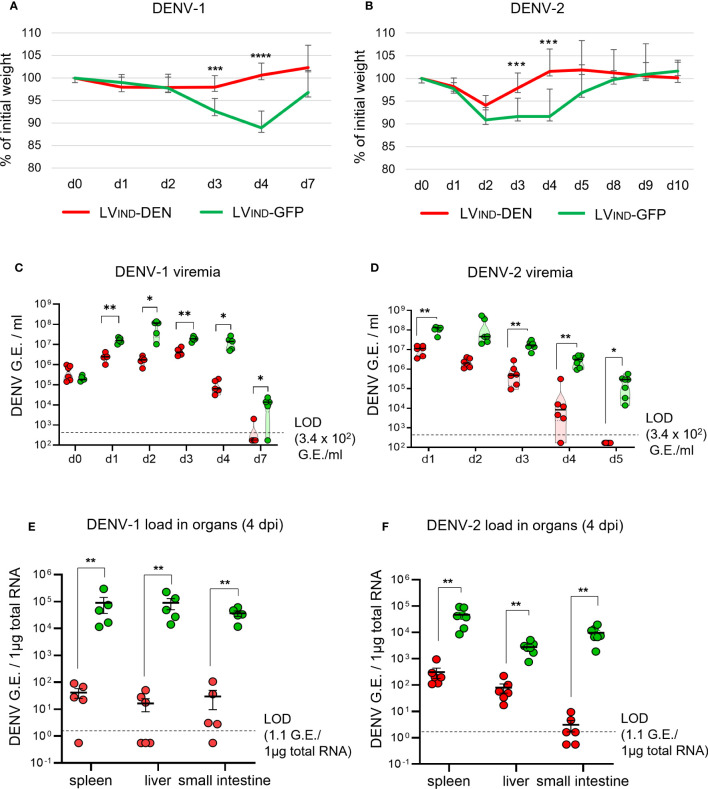
Protective potential of a single dose of LV-DEN against DENV-1 and DENV-2. IFNAR-BL6 mice (*n* = 5-6/group) received i.m. LV_IND_-DEN or LV_IND_-GFP and one month post-immunization were inoculated i.v. with 1 × 10^7^ FFU/mouse of DENV-1 **(A, C, E)** or 2 × 10^6^ FFU/mouse of DENV-2 **(B, D, F)**. Results were pooled from two independent experiments performed under the same conditions, after verification that no statistically significant differences were detected in the viremia levels and weight loss average between the LV_IND_-GFP control groups of the two experiments. **(A, B)** The percentage of the initial weight was determined for individual mice. Represented are the mean ± standard deviation of these percentages. The statistical significance of the differences between groups was evaluated by a two-tailed unpaired t-test (*** p < 0.01 and **** p < 0.0001). **(C, D)** Viremia expressed as genome equivalents (G.E.)/ml of plasma. **(E, F)** viral loads in the organs at 4 dpi, expressed as G.E./ 1µg of total RNA. LOD = limit of detection. The statistical significance of the differences between groups was evaluated by unpaired non-parametric Mann-Whitney test (* p < 0.05 and ** p < 0.01).

IFNAR-BL6 mice (*n* = 5-6/group) were immunized with a single i.m. dose of LV_IND_-DEN or LV_IND_-GFP. One month later, mice were inoculated with 1 × 10^7^ FFU/mouse of DENV-1 (strain KDH0026A) or 2 × 10^6^ FFU/mouse of DENV-2 (strain S221). No visible symptoms were detected in the challenged mice except for the weight loss that was observed in all groups during the first 2 days post-inoculation (dpi) (**Figures 5A, B**). All LV_IND_-DEN-vaccinated mice significantly regained weight between 3 and 4 dpi. In contrast, the LV_IND_-GFP-injected controls displayed a significantly delayed weight recovery, which generally occurred between 4 and 8 dpi for both viral infections. In LV_IND_-DEN-immunized mice, DENV-1 and DENV-2 viremia was significantly lower than in the control mice, from 1 and 2 dpi, respectively (**Figures 5C, D**). In LV_IND_-DEN-immunized mice, viremia declined significantly faster. Indeed, viremia was undetectable in most vaccinated mice at 7 and 5 dpi, in DENV-1- and DENV-2-inoculated mice, respectively, while both viruses were still largely detectable in the controls at these timepoints.

Both DENV-1 and DENV-2 loads in the spleen, liver, and small intestine were significantly lower at 4 dpi in LV_IND_-DEN-immunized mice (**Figures 5E, F**). Similar differences in the mean weight and viremia were detected between LV_IND_-DEN- and LV_IND_-GFP-immunized groups in A129 mice challenged with DENV-1 or DENV-2 (**Figure S3**). LV_IND_-DEN-immunized A129 mice showed statistically higher mean weight on 3 and 4 dpi and had significantly lower viremia on most dpi compared to the control mice.

**Figure 6:**
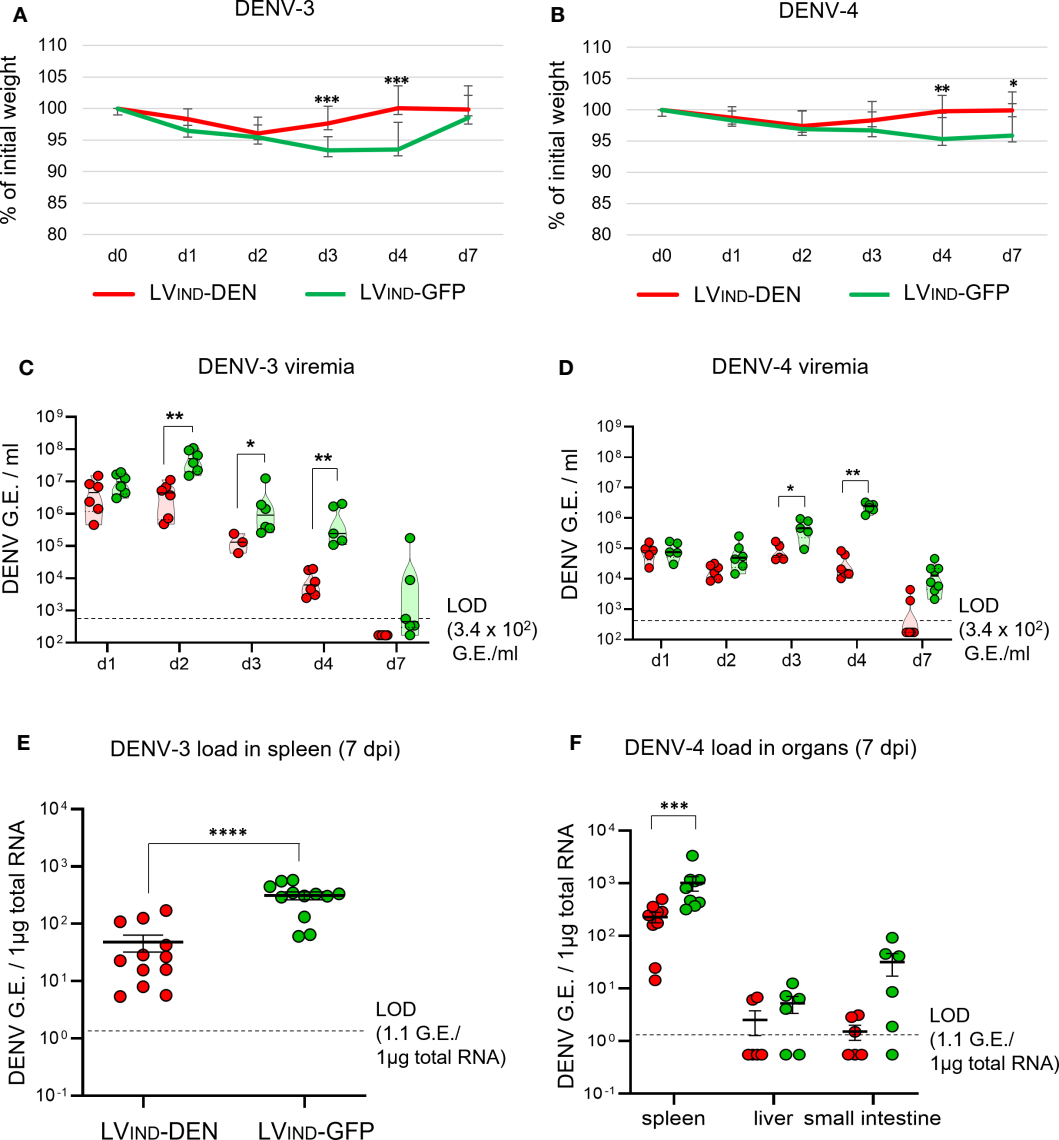
Protective potential of a single dose of LV-DEN against DENV-3 and DENV-4. IFNAR-BL6 mice (*n* = 6/group) received i.m. injection of LV_IND_-DEN or LV_IND_-GFP and were inoculated i.v. with 8 × 10^6^ FFU/mouse of DENV-3 two months post-immunization **(A, C, E)** or with 1 × 10^7^ FFU/mouse of DENV-4 a month post-immunization **(B, D, F)**. Results were pooled from two independent experiments performed in the same conditions after verification that no statistically significant differences were detected in the viremia levels and weight loss average between the LV_IND_-GFP control groups of the two experiments. **(A, B)** The percentage of the initial weight was determined for individual mice. The statistical significance of the differences between groups was evaluated by a two-tailed unpaired t-test (* p < 0.05, ** p < 0.01, and *** p < 0.001). **(C, D)** DENV-3 or DENV-4 viremia, expressed in G.E./ml of plasma. **(E)** DENV-3 load in the spleen and **(F)** DENV-4 loads in the spleen, liver, and small intestine, expressed as G.E./ 1µg of total RNA. Statistical significance of the differences between groups was evaluated by unpaired non-parametric Mann-Whitney test (* p < 0.05, ** p < 0.01, *** p < 0.001, and **** p < 0.0001).

To further evaluate the protective potential of LV_IND_-DEN against DENV-3 and DENV-4, IFNAR-BL6 mice (*n* = 6/group) were vaccinated i.m. with a single dose of LV_IND_-DEN or LV_IND_-GFP. Mice were inoculated with 8 × 10^6^ FFU/mouse of DENV-3 (strain PaH881/88) at 2 months post-immunization or with 1 × 10^7^ FFU/mouse of DENV-4 (strain ThD4_0087_77) 1 month post-immunization. The later post-immunization DENV-3 challenge was chosen to apply a more stringent condition for vaccine evaluation. Similar to DENV-1 and DENV-2 serotypes, DENV-3 and DENV-4 induced no particular symptoms in IFNAR-BL6 mice, except for the weight loss that was observed during the first 2 dpi. The mean weight of the LV_IND_-DEN-immunized and DENV-3-challenged mice was significantly higher than that of the control mice at 3-4 dpi (**Figure 6A**), and their viremia was significantly lower from 2 to 4 dpi (**Figure 6C**). Viremia was still detectable at 7 dpi in 4 of 6 control mice, but not in the LV_IND_-DEN-immunized mice. Furthermore, viral loads in the spleen of LV_IND_-DEN-immunized mice were significantly lower than in the control mice at 7 dpi (**Figure 6E**).

In the case of DENV-4 inoculation, significant regain of weight was observed at 4 and 7 dpi in LV_IND_-DEN-vaccinated mice (**Figure 6B**). The latter had generally lower viremia, starting at 2 dpi, with significant reductions observed at 3 and 4 dpi, compared to the control group (**Figure 6D**). Similar to the results obtained with DENV-3, DENV-4 viremia was detectable at 7 dpi in only 2 of 8 animals in the LV_IND_-DEN-immunized mice, while it was readily detectable in all LV_IND_-GFP-injected mice. Significantly lower viral loads were detected in the spleen of LV_IND_-DEN-vaccinated and DENV-4-challenged mice. Viral loads in the liver and small intestine were also lower in the LV_IND_-DEN-immunized mice, even if the differences did not reach statistical significance (**Figure 6F**).

Altogether, based on (i) faster weight recovery, (ii) significantly lower viremia, (iii) earlier viral clearance, and (iv) reduced viral loads in peripheral organs, the protection assays in both IFNAR-BL6 and A129 mice established the LV_IND_-DEN protective potential against the four DENV serotypes.

### Protective potential of a prime-boost LV-DEN vaccination regimen against DENV-2

A129 mice (*n* = 12/group) were primed i.m. with LV_IND_-DEN and boosted i.m. with LV_NJ_-DEN 2 months later. As indicated above, the use of distinct VSV-G pseudotyped LV in prime and boost was to avoid possible anti-VSV-G immunity that could reduce the boost’s efficacy. The control group received the same doses of LV-GFP. At 1 month post-boost, mice were inoculated with 1 × 10^7^ FFU/mouse of DENV-2 (strain S221).

**Figure 7:**
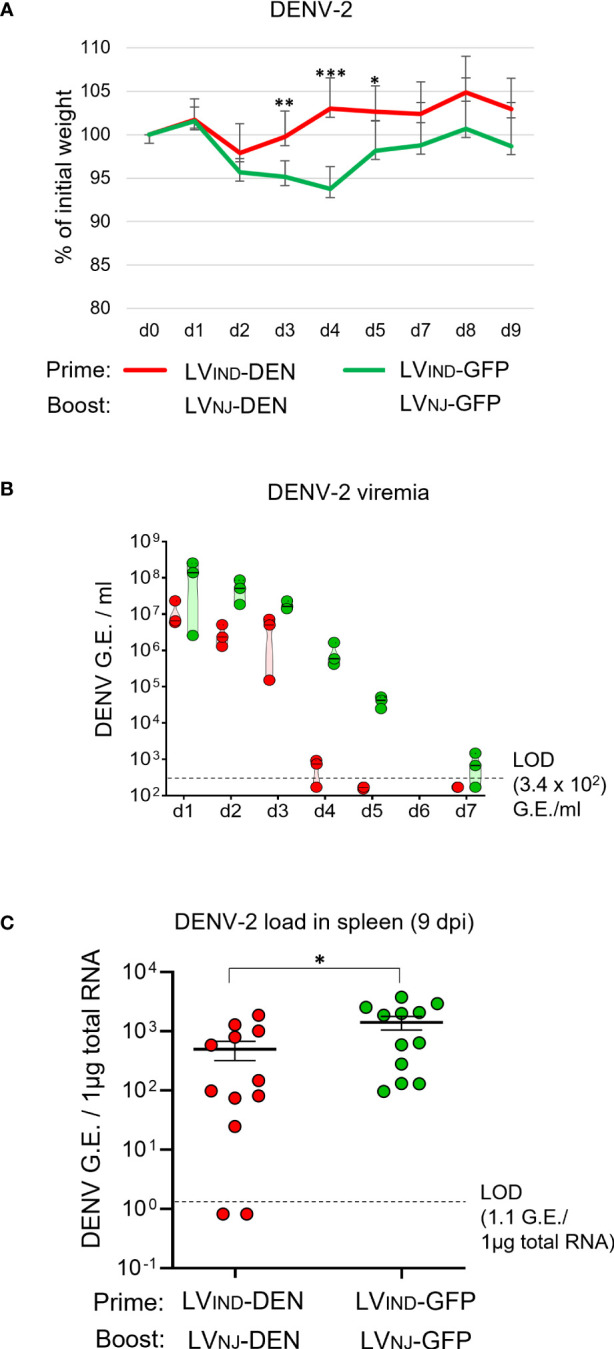
Protective potential of LV-DEN prime-boost against DENV-2. A129 mice (*n* = 12/group) received i.m. injection of LV_IND_-DEN or LV_IND_-GFP and were boosted i.m. 2 months later with LV_NJ_-DEN and LV_NJ_-GFP, respectively. A month post-boost, mice were inoculated i.v. with 1 × 10^7^ FFU/mouse of DENV-2. **(A)** The percentage of the initial weight was determined for individual mice. The statistical significance of the differences between groups was evaluated by a two-tailed unpaired t-test (* p < 0.05, ** p < 0.01, and *** p < 0.001). **(B)** Viremia expressed as G.E./ml of plasma, and **(C)** spleen viral load, expressed as G.E./ 1µg of total RNA. The statistical significance of the differences between groups was evaluated by unpaired non-parametric Mann-Whitney test (* p < 0.05).

All challenged mice of both groups displayed early weight loss (**Figure 7A**) as well as ruffled fur, which was not observed in DENV-infected IFNAR-BL6 mice. LV-DEN-primed-boosted mice regained weight earlier than the control mice with the significant differences recorded on 3 to 5 dpi (**Figure 7A**). The appearance of ruffled fur in the challenged mice generally correlated with weight loss and became less noticeable when mice began to regain weight. Lower mean viremia was detected in LV-DEN-primed-boosted mice from 2 dpi (**Figure 7B**). Similar to the protection results with a single dose immunization in A129 mice, the DENV-2 viremia in LV-DEN-primed-boosted mice decreased faster than in the control mice and was undetectable from 5 dpi (**Figure 7C**, **Figure S3**). Viral loads in the spleen of LV-DEN-immunized mice were significantly lower than in the control group. Overall, the levels of protection provided by the prime-boost regimen was comparable to those recorded after a single-dose immunization (**Figure S3**). Although the differences in viremia and viral load between the groups following prime-boost immunization in A129 mice were less pronounced than those observed in IFNAR-BL6 mice, this could be related to higher resistance of A129 mice to DENV infection.

### Early activation of antigen-specific CD8^+^ T cells in LV-DEN-vaccinated mice correlates with protection

**Figure 8:**
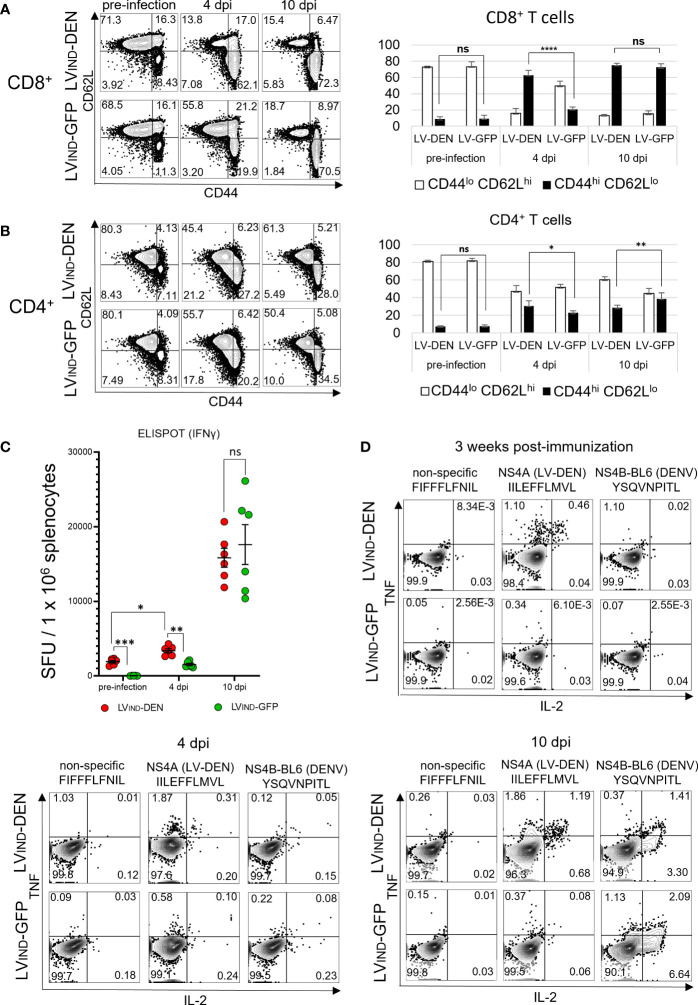
T-cell status in LV-DEN-vaccinated mice, before and after DENV-2 challenge. IFNAR-BL6 mice (*n* = 6/group) received i.m. injection of LV_IND_-DEN or LV_IND_-GFP and 1 month later were challenged i.v. with 2 × 10^6^ FFU/mouse of DENV-2. Expression of CD44 and CD62L markers by CD8^+^ **(A)** or CD4^+^ **(B)** T cells, collected before and after the challenge. Cytometry was performed on splenocytes of all individual mice (*n* = 6/group) and figures of one representative animal from each group are shown. Gating on **(A)** CD8^+^ (left panel) and **(B)** CD4^+^ (left panel) cells to identify populations of naive (CD44^lo^ CD62L^hi^) or activated (CD44^hi^ CD62L^lo^) T cells. Percentage of naive or activated cells versus **(A)** CD8^+^ (right panel) and **(B)** CD4^+^ (right panel) T cells before and after the challenge. Figures show mean counts from each group of animals (*n* = 6). Statistical significance of differences between groups of T cells expressing the selected markers was determined by a one-way ordinary ANOVA test with Tukey corrections for multiple comparisons (ns, non-significant, * p < 0.05, ** p < 0.01, and **** p < 0.0001). **(C)** Frequencies of IFNγ^+^ T splenocytes at day 21 post-immunization before the challenge and at 4 and 10 dpi, analyzed by IFNγ ELISPOT. Splenocytes of individual mice were re-stimulated with a peptide pool composed of seven peptides: six that were included in the DEN poly-antigen and one DENV-specific peptide that was not included in the DEN poly-antigen but was used for re-stimulation to take into account non-vaccine specific response to DENV infection (**Table S1**). Statistical significance of the differences between groups was evaluated by one-way Welch ANOVA test with Dunnett’s T3 corrections for multiple comparisons (ns, non-significant, * p < 0.05, ** p < 0.01, and *** p < 0.001). **(D)** ICS for TNF and IL-2 was performed on pooled CD8^+^ T splenocytes (*n* = 6/group) stimulated with individual peptides.

To characterize the dynamics of T-cell activation in LV-DEN-vaccinated mice following DENV inoculation, LV_IND_-DEN- or LV_IND_-GFP-injected mice were left unchallenged or were challenged 1 month later with 2 × 10^6^ FFU/mouse of DENV-2. Splenocytes from immunized mice were collected at day 21 post-immunization before the mice were challenged or at 4 and 10 dpi after the mice were challenged. T splenocytes were studied phenotypically for the expression of CD44 and CD62L T-cell markers and were analyzed functionally by ELISPOT and ICS (**Figure 8**).

At day 21 post-vaccination, the percentage of CD44^hi^ CD62L^lo^ activated cells in the CD8^+^ T subset was low and similar in LV_IND_-DEN-immunized mice and the control mice (**Figure 8A**). However, at 4 dpi, the percentages of these cells markedly increased in LV-DEN-immunized mice compared to the LV_IND_-GFP-injected and challenged group. No difference was recorded at 10 dpi when the percentages of CD44^hi^ CD62L^lo^ cells in the CD8^+^ T subset were equally high in both challenged groups (**Figure 8A**). This result indicates that LV_IND_-DEN vaccination favors early CD8^+^ T-cell mobilization after viral infection.

Statistically significant increases were also recorded in the percentages of CD44^hi^ CD62L^lo^ activated cells in the CD4^+^ T subset of LV_IND_-DEN and control groups at 4 dpi and 10 dpi (**Figure 8B**), albeit to a lesser extent compared to the CD8^+^ T subset. This suggests a more activated phenotype in the antigen-specific CD8^+^ T cell effectors, which are known to control viral infections.

As determined by ELISPOT, by 4 dpi, the frequencies of IFNγ^+^ T splenocytes in the LV-DEN-immunized mice increased significantly compared to the pre-infection stage and were also higher than in the LV-GFP-injected mice (**Figure 8C**). The frequencies of IFNγ^+^ T splenocytes in both the LV-DEN or LV-GFP-injected groups further increased by 10 dpi in a similar manner. This result further confirmed that LV_IND_-DEN vaccination favors early activation of antigen-specific CD8^+^ T-cells after viral infection.

In the ICS assay, CD8^+^ T splenocytes of LV_IND_-DEN-vaccinated mice responded to the *in vitro* stimulation with the NS4A peptide (IILEFLMVL) at day 21 post-immunization (**Figure 8D**). At 4 dpi, this response increased in the vaccinated mice compared to the controls. In comparison, at 4 and 10 dpi, but not after immunization alone, comparable percentages of cytokine-producing CD8^+^ T cells were detected in the two groups against NS4B (YSQVNPITL), an H-2^b^ restricted DENV-derived peptide (20) that was not included in the DEN poly-antigen and was thus used to detect cytokine expression induced exclusively by the challenge (**Figure 8D**). These data show an early DEN-specific CD8^+^ T-cell response in LV_IND_-DEN-immunized mice, which is associated with a decrease in viremia. In parallel, activation of naïve CD8^+^ T cells in response to DENV infection, which was observed in both groups of mice, occurred later.

Altogether, these results indicate that LV-DEN immunization induces rapid activation and mobilization of specific CD8^+^ T cells, characterized by CD44^hi^ and CD62^lo^ phenotype and IFNγ/TNF/IL-2 expression, early after DENV challenge.

### LV-DEN-induced protection is mediated by CD8^+^ T cells

**Figure 9:**
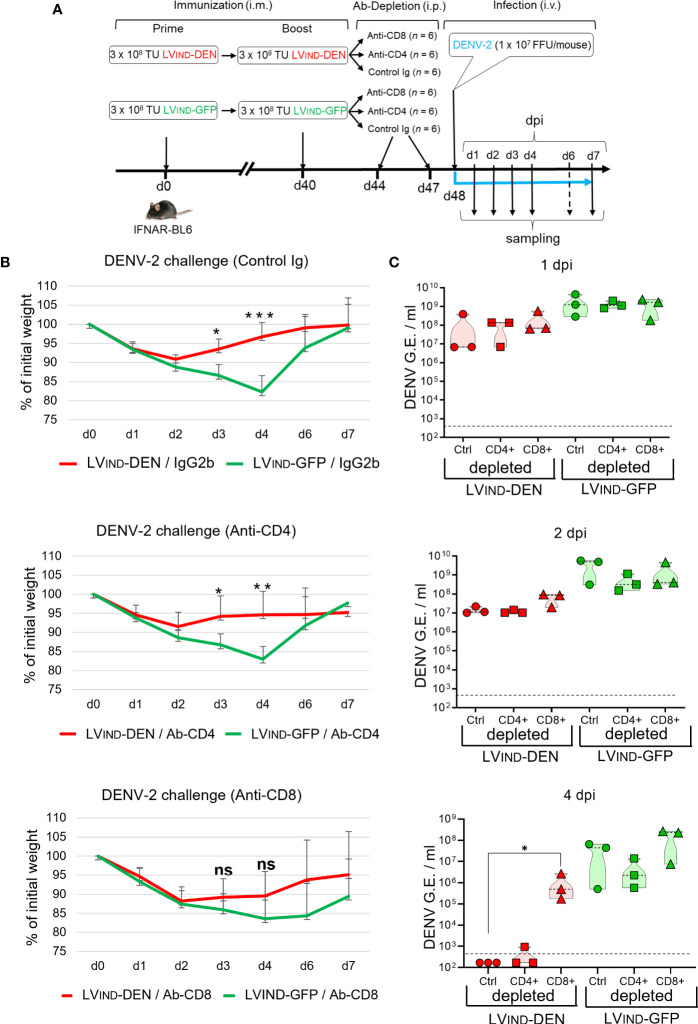
Role of CD8^+^ T cells in LV-DEN-induced protection. **(A)** Outline of the experiment. IFNAR-BL6 mice were primed i.m. at day 0 with LV_IND_-DEN or LV_IND_-GFP and boosted i.m. at day 40 with the same vectors, respectively. At day 8 post-boost, mice were inoculated i.v. with 1 × 10^7^ FFU/mouse of DENV-2. Prior to being challenged (at days 44 and 47) mice of each group (*n* = 6) were injected i.p. with 250 µg/mouse of either a control IgG2b isotype, anti-CD4, or anti-CD8 mAb. **(B)** Percentage of the initial weight and **(C)** viremia determined in individual mice. Blood samples were taken on alternating days from the subgroups of three mice to comply with the ethical protocol of animal experiments. Statistical significance of the differences between groups was evaluated by unpaired non-parametric Mann-Whitney test or Anova test for weight and viremia differences, respectively (ns, non-significant, * p < 0.05, ** p < 0.01, *** p < 0.001).

To assess the contribution of CD4^+^ and CD8^+^ T-cell subsets in the LV_IND_-DEN-induced protection, selective antibody-mediated depletion of these subsets was performed in vaccinated and challenged IFNAR-BL6 mice (**Figure 9A**). Mice (*n* = 6/group) were primed at day 0 and boosted at day 40 with LV-DEN or LV-GFP, prior to i.v. challenge at day 48 with 1 × 10^7^ FFU/mouse of DENV-2. Before the virus inoculation, mice were injected intra-peritoneally with anti-CD8, anti-CD4 mAb, or a rat IgG2b control isotype. The kinetics of weight loss and recovery in mice injected with anti-CD4 mAb or the control Ig isotype followed the pattern previously observed in mice untreated with mAb. In all groups a temporary weight loss was observed from 2 dpi, followed by a faster weight recovery in LV_IND_-DEN-immunized mice. In contrast, weight recovery of LV_IND_-DEN-immunized mice depleted of CD8^+^ T cells was delayed and no significant difference with the control group was detected in these mice at any time post-challenge (**Figure 9B**). Furthermore, a significant drop in viremia was observed in LV_IND_-DEN-immunized mice treated with either anti-CD4 mAb or control Ig isotype at 4 dpi, while the viremia in CD8^+^ T-cell depleted mice remained high and was similar to that detected in the LV_IND_-GFP-injected group (**Figure 9C**). Therefore, the protection provided by LV_IND_-DEN vaccination is mediated by CD8^+^ effector T cells without a sizeable contribution of CD4^+^ T cells.

## Discussion

Clinical trials with the first currently licensed tetravalent DENV Dengvaxia vaccine, currently licensed in 20 countries, demonstrated that this vaccine is not equally effective against different DENV serotypes and has ≈ 60% overall efficacy (8). The efficacy of Dengvaxia was significantly higher in DENV-immune than in the naïve subjects, suggesting that the vaccine mimics a primary DENV infection and may potentially increase vulnerability to subsequent DENV infections due to ADE. The vaccine has also been associated with higher hospitalization incidence rates and more severe disease in children younger than 9 years old (59) and is currently only recommended to DENV-immune individuals aged from 9 to 45 years (60, 61). Dengvaxia is a live-attenuated recombinant vaccine that expresses structural proteins of four DENV serotypes in the backbone of the yellow fever virus vaccine strain. Thus, the lack of protection could be attributed to the absence of DENV-specific T cells that recognize protective epitopes predominantly located in NS proteins.

Most of the currently used vaccine development strategies against DENV employ either live-attenuated DENV viruses (TAK-003 and TV-003/005) or subunit-, DNA/RNA-, or viral vector-based vaccines using adenovirus-, vaccinia virus-, and alphavirus-based vectors, and targeting DENV structural surface proteins (62). Several approaches to target DENV NS proteins have also been explored. However, they are mainly based on NS1, a secreted DENV protein recognized by both humoral and cellular immune responses (62, 63). Although several studies showed protective effect of such vaccine candidates in murine models, the beneficial effect of vaccines expressing NS1 in human trials remains controversial. Anti-NS1 antibodies recognize human fibrinogen and cross-react with thrombocytes and endothelial cells, leading to enhanced vascular permeability and increased secretion of pro-inflammatory cytokines (64, 65). 

Previous studies have reported that NS3, NS4B, and NS5 proteins are the main targets of cytotoxic T-cell responses in humans with potential cross-protective effects against distinct DENV serotypes (12, 14, 66). Our results demonstrate that immunization with an LV encoding the DEN T-cell poly-antigen including immunogenic regions of NS3, NS5, NS4B, and NS4A, induces highly significant protective effects against challenge with each of the four DENV serotypes in the preclinical murine model. LV-DEN-mediated protection is manifested in mice by faster post-challenge weight recovery, decreased viremia, and reduced viral loads in peripheral organs. Due to small quantities of blood collected from infected mice, mainly because of ethical considerations, we were unable to systematically measure the quantities of infectious virus by TCID50 titration method, in parallel with the RT-qPCR assay. However, measurement of infectious DENV titers in the samples of a few individual animals indicated the same range of difference in the viral titers between mice immunized with LV-DEN or LV-GFP as determined by the RT-qPCR. In the present study, the amounts of infectious DENV measured by TCID50 were approximately 10^4^-fold lower than the number of genome equivalents (G.E.) determined by RT-qPCR. This observation is in agreement with other previous studies (20, 67).

Selective depletion of CD4^+^ and CD8^+^ T cells showed that the protection is mediated through the action of CD8^+^ T cells. These vaccine-induced effector T cells are activated and mobilized after a DENV challenge, much earlier than the infection itself could induce. To our knowledge, this is the first demonstration of the protective efficacy provided by a viral vector that encodes a T-cell poly-epitope derived from DENV NS proteins and thus does not rely on the induction of neutralizing antibodies. No significant induction of the CD4^+^ T-cell response against the selected DEN antigen could be detected, likely because lentiviral vectors, like the vast majority of viral vectors, do not efficiently orient antigens to the MHC-II presentation pathway inside the transduced antigen-presenting cells (37, 68). In addition, the DEN antigen designed here was not secreted, therefore it was not taken up by bystander antigen-presenting cells. Although DENV-specific CD4^+^ T-cell responses were shown to be dispensable for controlling primary DENV infections in *ifnar-/-* mice, prior induction of such responses by immunization contributed to protection (21). In humans, DENV-specific CD4^+^ T cells could play important roles by assisting CD8^+^ T-cell and B-cell antiviral responses as well as contributing to protection via direct cytotoxic action (69, 70). Thus, it could be desirable to enhance the engagement of CD4^+^ T-cell response via antigen and/or vector design.

While another lentiviral vector design that can efficiently target antigens to the MHC-II presentation pathway is currently possible (68), it requires a significant reduction of the antigen’s size, which is likely to compromise the magnitude of cross-serotype MHC class I response against DENV. However, even if DEN antigen is not specifically targeted by CD4^+^ T-cell response, such response is likely to be generated against lentiviral vector itself and could improve the quality and longevity of DEN-specific CD8^+^ T cells by providing necessary co-stimulatory signals, particularly in the case of prime-boost immunizations. Although a single prime-boost immunization/protection experiment performed in this study did not suggest significant improvement in protection compared to a single-dose immunization, this question will be addressed in the future.

Previous studies demonstrated that immunization of *ifnar^-/-^* mice with adjuvanted DENV-2-specific peptides followed by a homologous challenge resulted in an approximately 10-fold decrease in viremia, which was totally abrogated by depletion of CD8^+^ T cells (20). In another study, immunization of *ifnar^-/-^* mice, transgenic for human allele HLA-B*07:02, with DENV-2 NS3-derived peptides or a mixture of DENV-1/3/4-derived cross-reactive peptides, formulated in adjuvant, led to similar decreases of DENV-2 and DENV-3 viremia (26). More recently, immunization with an mRNA vaccine encoding for DENV-1-derived HLA class I poly-antigen resulted in a significant reduction of DENV-1 viremia as assessed in *ifnar* wild-type mice, which are transgenic for several HLA-alleles and sensitized for DENV infection by anti-IFNα mAb pre-treatment (71). Remarkably, a differential decrease in viremia was observed for different HLA alleles. Up to 10-fold decrease in viremia was seen in mice transgenic for HLA-A*24:02 and HLA-B*35:01, while no decrease was observed in mice transgenic for HLA-A*02:01 (71), which logically suggests the contribution of immunogenetics linked to the HLA haplotype in vaccine efficacy, but which is certainly minimized in humans because of the availability of several HLA molecules, compared to a transgenic mouse expressing only one.

Finally, in another immunocompetent mouse model of intracranial DENV challenge, immunization with a mixture of recombinant C proteins of four DENV serotypes resulted in a differential decrease in viremia for various DENVs, ranging from 36 to 413 folds, with the highest reduction seen for DENV-4. This activity was again attributed to T-cell responses, as no anti-DENV antibodies were detected post-immunization (72). Experimental studies of DENV infection are complicated by the absence of immunocompetent small animal models that could mimic the main characteristics of dengue disease (73). Another limitation of using mouse models for the evaluation of human T-cell responses induced by vaccine candidates is that DENV-specific T cells of humans and mice do not target the same epitopes. For instance, while NS3 protein was shown to be one of the major targets of CD8^+^ T-cell responses in humans, only very few known murine MHC-I epitopes are located on NS3. It is also important to note that the DEN poly-antigen was designed to include a maximal number of human epitope clusters, while only 41% of the known murine epitopes were included to enable the validation of proof of concept (**Figure S2**). This, along with the reportedly less efficient transduction of mouse cells by HIV-based lentiviral vectors in comparison to human cells (74, 75), suggest that the murine models may underestimate the protection provided by LV-DEN.

Although the development of a T-cell vaccine minimizing the risk of ADE is attractive, this approach presents some potential difficulties. The variability of T-cell responses in the global human population represents a major challenge. In humans immunized with live-attenuated DENV vaccine candidates, T-cell responses could be classified into four distinct types: (i) broad strong responses associated with HLA-B*07:02 and HLA-B*35:01, (ii) broad weak responses associated with HLA-A*26:01, (iii) strong narrow responses associated with HLA-B*40:01, and (iv) narrow weak responses associated with HLA-A*01:01 and HLA-A*24:02 (12). This disparity indicates that the quality of vaccine-induced T-cell responses and the efficiency of protection could be dependent on immuno-genetic characteristics and notably the HLA haplotype.

In conclusion, our data provide proof of concept for the use of lentiviral vectors expressing DENV NS-derived T-cell polyepitopes for induction of DENV-specific CD8^+^ T-cell immunity. These responses are able to significantly protect mice against four DENV serotypes, resulting in decreased viral loads, reduced morbidity, and faster weight recovery of immunized animals.

## Data availability statement

The original contributions presented in the study are included in the article/**Supplementary Material**. Further inquiries can be directed to the corresponding author.

## Ethics statement

The animal study was reviewed and approved by Safety, Animal Care and Use Committee (Institut Pasteur, France) and French Ministry of High Education and Research.

## Author contributions

Study concept and design: KN, MB, LM, and PC; acquisition of data: KN, PA, and PS; construction and production of lentiviral vectors and technical support: PS, FM, AN, and CB; analysis and interpretation of data: KN, MB, LM, and PC; and drafting of the manuscript: KN, LM, and PC. All authors contributed to the article and approved the submitted version.

## Supplementary material

Supplementary Figure 1 Alignment of the amino acid sequences included in the DEN poly-antigen. Antigenic regions were selected from NS3 **(A)**, NS4A, 2K, and NS4B **(B)** and NS5 **(C)** proteins. The first sequence in the alignment (DENV1-4_cons) is a 75% majority consensus sequence of 4 DENV serotypes, created on the basis of the individual consensus sequences of each serotype (DENV1_cons, DENV2_cons, DENV3_cons, and DENV4_cons). Sequence of DEN poly-antigen is marked by the red color.

Supplementary Figure 2 Sequence of DEN poly-antigen. **(A)** Arrangement of the individual protein fragments originating from NS DENV proteins in the DEN poly-antigen. The amino acid linkers (L1 to L10), connecting different regions, were designed to avoid generation of non-specific MHC-I epitopes. **(B)** Protein sequence of DEN poly-antigen. Regions predicted to contain MHC-I epitopes of H-2^b^ mice are indicated in red.

Supplementary Figure 3 Protective potential of a single dose of LV-DEN against DENV-1 and DENV-2. A129 mice (n = 10/group) received i.m. injection of LV_IND_-DEN or LV_IND_-GFP and one month later were inoculated i.v. with either 1 × 10^7^ FFU/mouse of DENV-1 **(A, C)** or 5 × 10^5^ FFU/mouse of DENV-2, depending on what the titer of the viral stock allowed **(B, D)**. **(A, B)** Percentage of the initial weight was determined for individual mice. Represented are mean ± standard deviation of these percentages. **(C, D)** Viremia expressed as genome equivalents (G.E.)/ml of plasma. LOD = limit of detection. Statistical significance of the differences between groups was evaluated by unpaired non-parametric Mann-Whitney test (* p < 0.05, ** p < 0.01, *** p < 0.001).
