# Supplementary material for: Preclinical proof of concept of a tetravalent lentiviral T-cell vaccine against dengue viruses
Source: Front Immunol. 2023 Aug 15;14:1208041. doi: 10.3389/fimmu.2023.1208041 (PMC10466046; doi:10.3389/fimmu.2023.1208041)
Supplement: Supplementary file 1 [file Table_1.docx]

**Table S1.** **Peptides used to assess immunogenicity of LV-DEN in H-2^b^ mice.**

| **Pepride origin** | **Pool name** | **Used in Elispot (Figure 3)** | **Used in ICS (Figure 4)** | **Used in Elispot (Figure 8C)** |
| --- | --- | --- | --- | --- |
| DEN poly-antigen | NS3-3 | KPRWLDARIYSDPLA | KPRWLDARIYSDPLA |  |
|  |  | LDARIYSDPLALKEF | LDARIYSDPLALKEF |  |
|  |  | IYSDPLALKEFKEF | IYSDPLALKEFKEF |  |
|  | NS4A | IILEFFLMVL | IILEFFLMVL | IILEFFLMVL |
|  | NS4B-1 | LRPASAWTLYAVATT | LRPASAWTLYAVATT |  |
|  |  | SAWTLYAVATTFVTP | SAWTLYAVATTFVTP |  |
|  |  | LYAVATTFVTPMLRH | LYAVATTFVTPMLRH |  |
|  |  | ATTFVTPMLRHTIEN | ATTFVTPMLRHTIEN |  |
|  | NS4B-2 | VLMMRTTWALCEALT |  |  |
|  |  | RTTWALCEALTLATG |  |  |
|  |  | ALCEALTLATGPIST |  |  |
|  |  | ALTLATGPISTLWEG |  |  |
|  |  | ATGPISTLWEGSPGK |  |  |
|  |  | ISTLWEGSPGKFWNT |  |  |
|  |  | WEGSPGKFWNTTIAV |  |  |
|  |  | PGKFWNTTIAVSMAN |  |  |
|  |  | WNTTIAVSMANIFRG |  |  |
|  |  | IAVSMANIFRGSYLA |  |  |
|  |  | MANIFRGSYLAGAGL |  | GSYLAGAGL |
|  |  | FRGSYLAGAGLAF |  |  |
|  | NS5-2 | SSMVNGVVKLLTKPW | SSMVNGVVKLLTKPW | SSMVNGVVKLLTKPW |
|  |  | NGVVKLLTKPWDVVP |  |  |
|  |  | KLLTKPWDVVPMVTQ |  |  |
|  |  | KPWDVVPMVTQMAMT |  |  |
|  |  | VVPMVTQMAMTDTTP |  |  |
|  |  | VTQMAMTDTTPFGQQ | VTQMAMTDTTPFGQQ | VTQMAMTDTTPFGQQ |
|  |  | AMTDTTPFGQQRVF |  |  |
|  | NS5-3 | GSRAIWYMWLGARFL |  | RAIWYMWL |
|  |  | IWYMWLGARFLEFEA |  |  |
|  |  | WLGARFLEFEALGFL |  |  |
|  | NS5-4 | RETACLGKSYAQMWS |  |  |
|  |  | CLGKSYAQMWSLMYF | KSYAQMWSL | KSYAQMWSL |
|  |  | SYAQMWSLMYFHRRD |  |  |
|  |  | MWSLMYFHRRDL |  |  |
| DENV | NS4B-BL6 |  |  | YSQVNPITL |
| YFV | YF-C |  | FIFFFLFNIL |  |

For primary analysis of immunogenicity by ELISPOT (Figure 3) overlapping 15-mer peptides that covered 7 predicted immunogenic regions were used. In subsequent analyses a selection of peptide pools that demonstrated the highest immunogenicity, i.e., NS4B-1 and NS3-3 and/or individual peptides containing MHC-I predicted epitopes were used. NS4B-BL6 is a known DENV-specific MHC-I epitope in H-2^b^ mice not expressed by DEN poly-antigen. This peptide was added to the pool of peptides used in ELISPOT (Figure 8C) to determine T-cell responses induced by the viral challenge, rather than immunization. YF-C is a YFV specific MHC-I epitope of H-2^b^ mice that was used as a negative control in the ICS assays.
