# Supplementary figures and images for: Preclinical proof of concept of a tetravalent lentiviral T-cell vaccine against dengue viruses
Source: Front Immunol. 2023 Aug 15;14:1208041. doi: 10.3389/fimmu.2023.1208041 (PMC10466046; doi:10.3389/fimmu.2023.1208041)

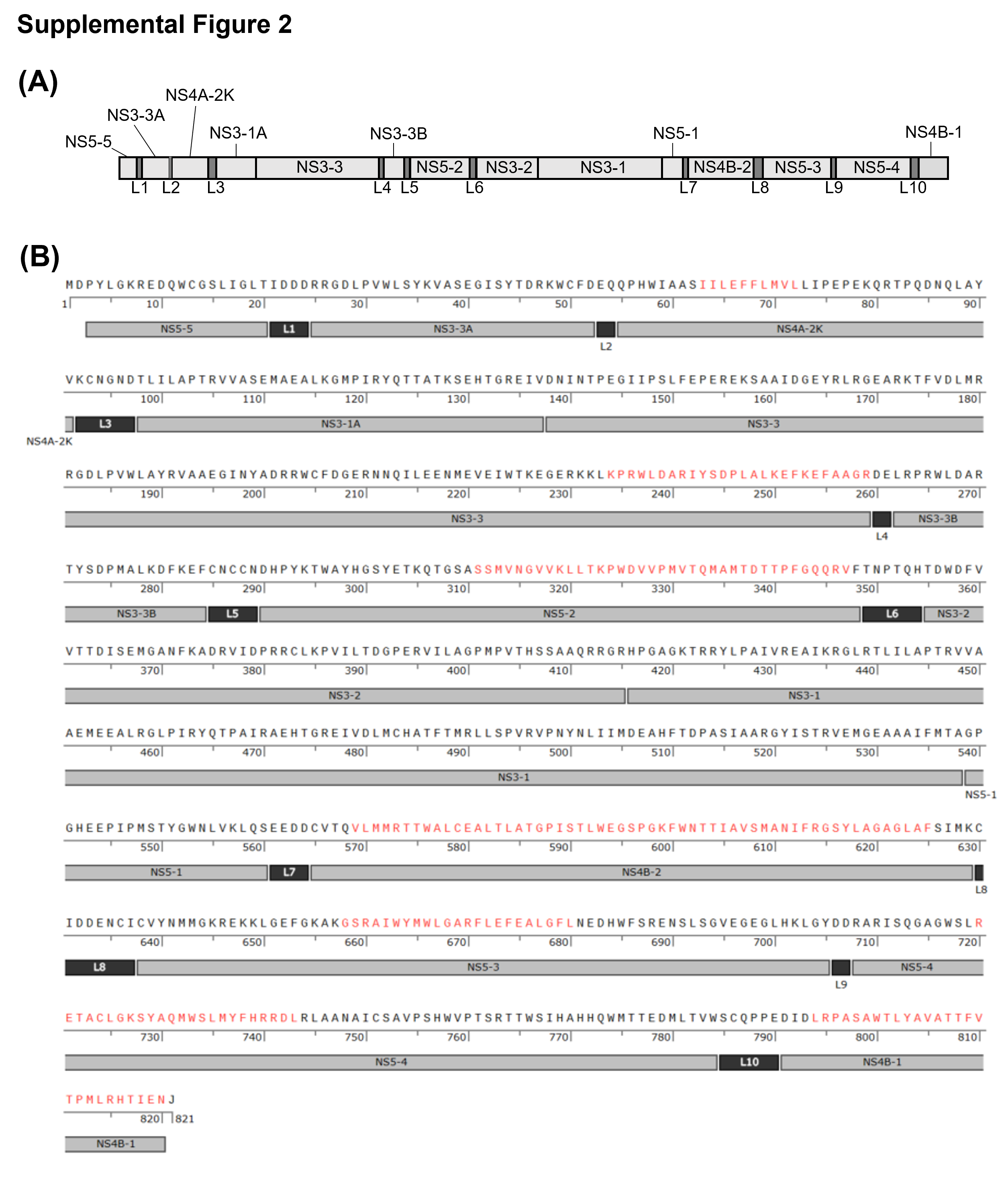

Supplement: Supplementary Figure 1 — Alignment of the amino acid sequences included in the DEN poly-antigen. Antigenic regions were selected from NS3 (A), NS4A, 2K, and NS4B (B) and NS5 (C) proteins. The first sequence in the alignment (DENV1-4_cons) is a 75% majority consensus sequence of 4 DENV serotypes, created on the basis of the individual consensus sequences of each serotype (DENV1_cons, DENV2_cons, DENV3_cons, and DENV4_cons). Sequence of DEN poly-antigen is marked by the red color. [file Image_1.tif]

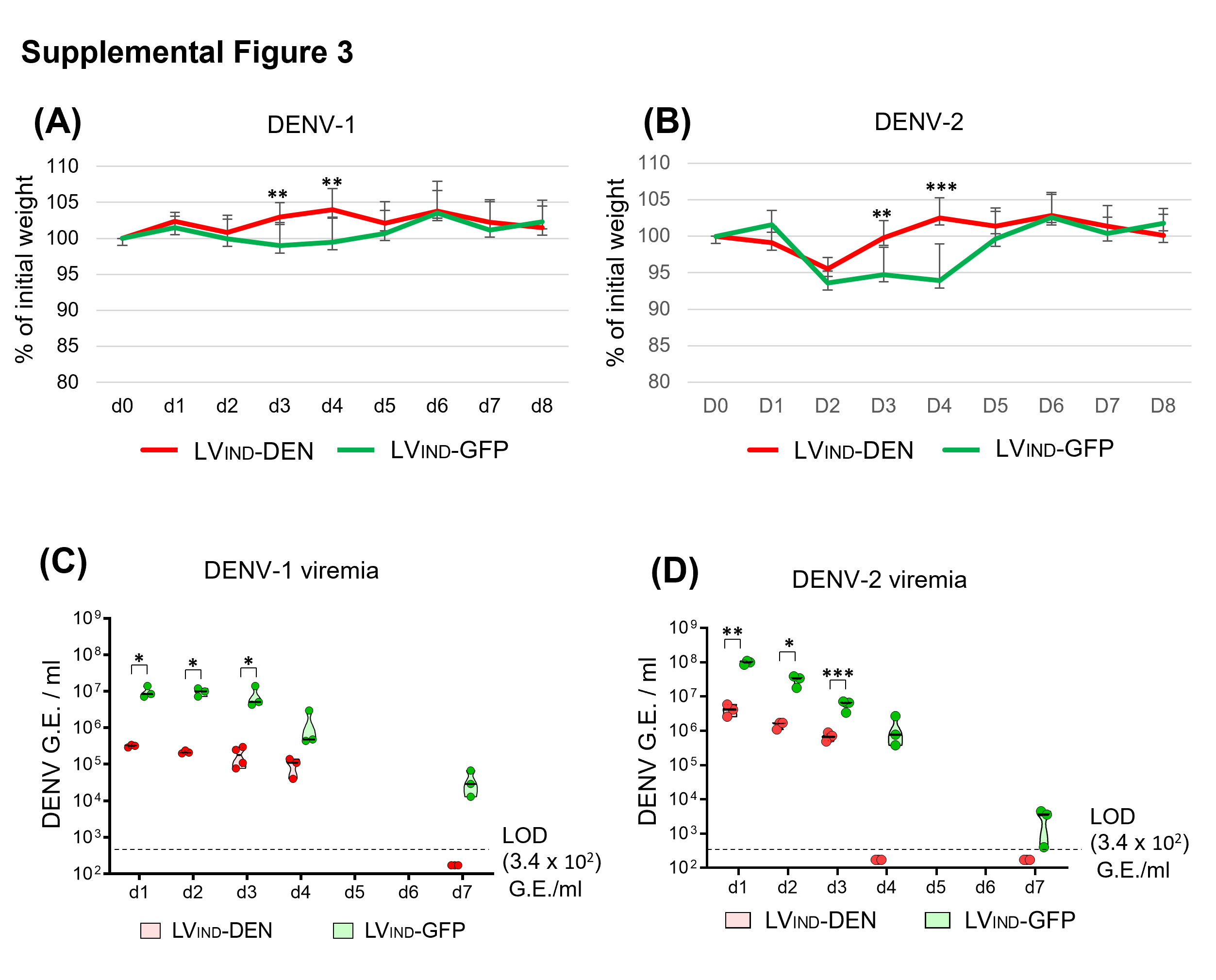

Supplement: Supplementary Figure 2 — Sequence of DEN poly-antigen. (A) Arrangement of the individual protein fragments originating from NS DENV proteins in the DEN poly-antigen. The amino acid linkers (L1 to L10), connecting different regions, were designed to avoid generation of non-specific MHC-I epitopes. (B) Protein sequence of DEN poly-antigen. Regions predicted to contain MHC-I epitopes of H-2b mice are indicated in red. [file Image_2.tif]

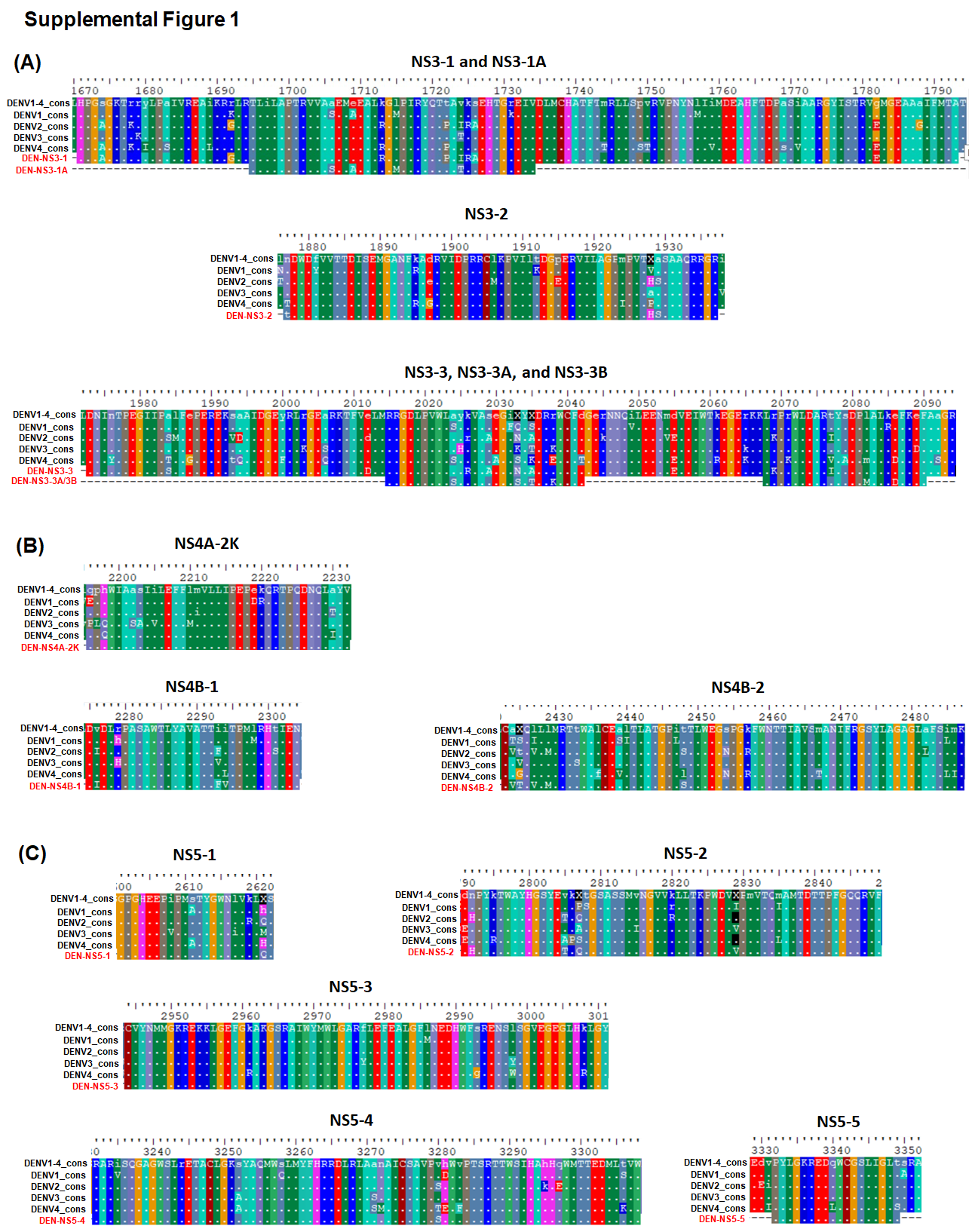

Supplement: Supplementary Figure 3 — Protective potential of a single dose of LV-DEN against DENV-1 and DENV-2. A129 mice (n = 10/group) received i.m. injection of LVIND-DEN or LVIND-GFP and one month later were inoculated i.v. with either 1 × 10^7^ FFU/mouse of DENV-1 (A, C) or 5 × 10^5^ FFU/mouse of DENV-2, depending on what the titer of the viral stock allowed (B, D). (A, B) Percentage of the initial weight was determined for individual mice. Represented are mean ± standard deviation of these percentages. (C, D) Viremia expressed as genome equivalents (G.E.)/ml of plasma. LOD = limit of detection. Statistical significance of the differences between groups was evaluated by unpaired non-parametric Mann-Whitney test (* p < 0.05, ** p < 0.01, *** p < 0.001). [file Image_3.tiff]
